# Motor Neuron Generation from iPSCs from Identical Twins Discordant for Amyotrophic Lateral Sclerosis

**DOI:** 10.3390/cells9030571

**Published:** 2020-02-28

**Authors:** Emily R. Seminary, Stephanie Santarriaga, Lynn Wheeler, Marie Mejaki, Jenica Abrudan, Wendy Demos, Michael T. Zimmermann, Raul A. Urrutia, Dominic Fee, Paul E. Barkhaus, Allison D. Ebert

**Affiliations:** 1 Department of Cell Biology, Neurobiology, and Anatomy, Medical College of Wisconsin, Milwaukee, WI 53226, USA; eseminary@mcw.edu (E.R.S.); SSANTARRIAGA@mgh.harvard.edu (S.S.); 2 Department of Neurology, Medical College of Wisconsin, Milwaukee, WI 53226, USA; lwheeler@mcw.edu (L.W.); mmejaki@mcw.edu (M.M.); dfee@mcw.edu (D.F.); pbarkhaus@mcw.edu (P.E.B.); 3 Bioinformatics Research and Development Laboratory, Genomic Sciences and Precision Medicine Center, Medical College of Wisconsin, Milwaukee, WI 53226, USA; pabrudan@mcw.edu (J.A.); wdemos@mcw.edu (W.D.); mtzimmermann@mcw.edu (M.T.Z.); rurrutia@mcw.edu (R.A.U.); 4 Clinical and Translational Science Institute and Department of Biochemistry, Medical College of Wisconsin, Milwaukee, WI 53226, USA; 5 Department of Surgery, Medical College of Wisconsin, Milwaukee, WI 53226, USA

**Keywords:** induced pluripotent stem cells, protein aggregation, glutamate toxicity, SOD1, C9orf72, sporadic ALS, familial ALS

## Abstract

Amyotrophic lateral sclerosis (ALS) is a complex neurodegenerative disorder characterized by the loss of the upper and lower motor neurons. Approximately 10% of cases are caused by specific mutations in known genes, with the remaining cases having no known genetic link. As such, sporadic cases have been more difficult to model experimentally. Here, we describe the generation and differentiation of ALS induced pluripotent stem cells reprogrammed from discordant identical twins. Whole genome sequencing revealed no relevant mutations in known ALS-causing genes that differ between the twins. As protein aggregation is found in all ALS patients and is thought to contribute to motor neuron death, we sought to characterize the aggregation phenotype of the sporadic ALS induced pluripotent stem cells (iPSCs). Motor neurons from both twins had high levels of insoluble proteins that commonly aggregate in ALS that did not robustly change in response to exogenous glutamate. In contrast, established genetic ALS iPSC lines demonstrated insolubility in a protein- and genotype-dependent manner. Moreover, whereas the genetic ALS lines failed to induce autophagy after glutamate stress, motor neurons from both twins and independent controls did activate this protective pathway. Together, these data indicate that our unique model of sporadic ALS may provide key insights into disease pathology and highlight potential differences between sporadic and familial ALS.

## 1. Introduction

Amyotrophic lateral sclerosis (ALS) is a fatal neurodegenerative disorder caused by the selective death of the upper and lower motor neurons (MNs). MN loss causes a progressive paralysis that affects a patient’s ability to move, speak, and breathe; ALS typically leads to death within 3 to 5 years of diagnosis. The vast majority of cases (approximately 90%) occur in the absence of a family history, denoted as sporadic ALS (sALS) [1]. However, familial ALS (fALS), linked to a mutation in one of the two dozen known causative genes [1], is most often studied due to the ease of experimental model generation. Still, given the diversity of normal functions that these genes have [2], it is unclear whether similar mechanisms contribute to MN death in fALS and whether these models accurately describe the sporadic conditions.

Protein aggregates can be found in virtually all ALS patients [3,4,5,6]. More than 90% of patients have aggregates of TDP-43, although SOD1, optineurin (OPTN), p62, and ubiquitin are also common [3,4,5,6]. Data support the idea that protein aggregation contributes to cell death by leading to oxidative stress and general cellular dysfunction through sequestering cytosolic proteins to the aggregates themselves [7,8,9,10]. Recent studies documenting liquid–liquid phase separation suggest that dynamic, soluble phase separation is a protective mechanism in the cell [11], but a more recent study demonstrated that phase-separated TDP-43 is more toxic to cells than hydrophobic aggregates [12]. Although the exact pathogenic role of the inclusions is unclear, protein aggregation is a key phenotype of ALS and requires more investigation across multiple genetic backgrounds. 

Induced pluripotent stem cells (iPSCs) represent a powerful tool to model a wide array of diseases as iPSCs can theoretically be generated from any individual regardless of health status or genetics. Like embryonic stem cells, iPSCs can be differentiated into virtually every cell type of the body [13], thereby making it possible to model sporadic diseases in vitro. However, without isogenic controls, it is not immediately clear which of the observed phenotypes in disease lines are due to the disease itself and not normal population variance. CRISPR/Cas9 technology has made generating isogenic controls relatively straightforward for fALS models, but this has remained elusive for sALS models.

In this study, we generated iPSCs from discordant identical twin brothers to model sALS in an isogenic manner. We found that the brothers have no pathogenic mutations in known ALS-causing genes, and that MNs generated from both twins exhibit high levels of insoluble proteins. Additionally, whereas MNs derived from iPSCs expressing pathogenic SOD1 or C9orf72 mutations alter protein burden in response to excess glutamate in a protein- and genotype-dependent manner, the MNs from the identical twins do not robustly change their aggregate levels after the same stress. They are, however, capable of inducing autophagic flux at levels seen in healthy control cells in response to excess glutamate, which is a mechanism that the SOD1 and C9orf72 MNs do not activate. Together, these data indicate that these novel iPSC lines may provide key insights into disease pathology and identify convergent and divergent mechanisms between fALS and sALS.

## 2. Materials and Methods

### 2.1. Human Sample Collection

Identical twin males discordant for ALS were identified from the ALS Multi-Disciplinary Clinic at Froedtert Hospital and consented to sample collection in accordance to the procedures outlined by IRB PRO00024167 (approved 29 September 2015). The affected individual was diagnosed with ALS with left leg onset at the age of 59 and collection occurred at age 61. The disease duration was 64 months from symptom onset.

### 2.2. Induced Pluripotent Stem Cells (iPSC) Reprogramming and Validation

Peripheral blood mononuclear cells (PBMCs) were isolated and reprogrammed as described previously [14] using the CytoTune-iPS 2.0 Sendai Reprogramming Kit (Life Technologies, Carlsbad, CA, USA). Individual clones were picked and expanded separately. Pluripotency was confirmed through qPCR for Oct4, Sox2, and Nanog; immunocytochemistry for Oct4, Sox2, Tra 1-81; and embryoid body (EB) formation and subsequent immunocytochemistry for α-fetoprotein (endoderm), smooth muscle actin (mesoderm), and Tuj1 (ectoderm), as described previously [15,16]. Two independent clones for each patient were used for further studies.

### 2.3. Whole Genome Sequencing

Whole genome libraries were constructed by standard automated methods using Illumina TruSeq technology. Quantitative Real-time PCR was used to assess the overall amount of DNA library. Samples were sequenced on the Illumina HiSeq 2500 (Illumina, San Diego, CA, USA) with 125 bp paired-end reads with an average depth of coverage of 43X. Raw sequencing data were converted to the fastq format by bcl2fastq conversion software v 1.8.4 (Illumina, San Diego, CA, USA) and were aligned to Human reference GRCh37 by the Burrows–Wheeler Alignment Minimal Exact Matches (BWA-MEM) algorithm v0.7.15 [17]. Variant calls were generated through the Genome Analysis Toolkit [18] v 3.7 Haplotype Caller according to current Best Practices recommendations (GATK, Broad Institute [19]). The variants were hard filtered using a minimum depth of 8 for the combined read depth across both samples.

### 2.4. Stem Cell Culture

Induced pluripotent stem cells (iPSCs) were maintained in Essential 8 (Life Technologies, Carlsbad, CA, USA) on Matrigel (Corning, Corning, NY, USA) coated 6-well plates (VWR, Radnor, PA, USA) and passaged every 4–6 days with Versene (Life Technologies, Carlsbad, CA, USA). Cultures were routinely tested for mycoplasm using the Mycoalert Detection Kit (Lonza, Basel, Switzerland). Two independent control lines [16,20], one N139K SOD1 line [21], one A4V SOD1 line (Coriell, Camden, NJ, USA), and two independent C9orf72 lines [22] were also used.

### 2.5. Motor Neuron Differentiation

Motor neurons (MNs) were generated as described previously [23] and were plated onto Matrigel-coated plates or glass coverslips. For glutamate toxicity experiments, cultures were treated with 100 µM glutamate for 96 h at four weeks of total differentiation.

### 2.6. Immunocytochemistry

Motor neurons (MNs) were fixed after four weeks of differentiation and stained as previously described [15]. Images were taken on an upright Nikon E400 microscope (Nikon, Minato City, Tokyo, Japan) with a 40× objective and a QCapture camera (QImaging, Tucson, AZ, USA). Neurite length was measured using the line trace function in NIS Elements only from individual cell bodies with identifiable projections. The number of neurites measured was consistent across each of the cell lines. Quantification was performed in NIS Elements (Nikon) by a blinded observer. A list of antibodies used can be found in Table 1.

### 2.7. Protein Isolation and Analysis

Protein was isolated using the Triton-X 100 fractionation method. Briefly, cell pellets were resuspended in a 1% Triton X-100 lysis buffer and briefly sonicated. Protein concentration was determined through a bicinchoninic acid (BCA) assay (Life Technologies, Carlsbad, CA, USA) according to manufacturer’s instructions. Insoluble protein was isolated via ultra-centrifugation at 45,000 rpm for 30 min and the resulting pellet was resuspended with 2% SDS. Insoluble protein was quantified using a BCA assay. 10 µg of soluble protein or 30–40 µL of insoluble protein were used for Western Blot analysis [15]. Blots were imaged with the Odyssey Scanner (Li-COR, Lincoln, NE, USA), and all soluble signals were quantified in Image Studio (Li-COR, Lincoln, NE, USA) and normalized to REVERT Total Protein Stain (Li-COR, Lincoln, NE, USA), according to manufacturer’s instructions. Representative images were converted to grayscale for figures. A list of antibodies used can be found in Table 1.

### 2.8. Statistical Analysis

A minimum of three independent experiments each with a minimum of three biological replicates were analyzed. The data were analyzed with a Student’s *t*-test or a one-way ANOVA with Tukey’s post test, as appropriate. *p* < 0.05 was considered significant.

## 3. Results

### 3.1. Generation of Induced Pluripotent Stem Cells (iPSCs) from Identical Twins Discordant for Amyotrophic Lateral Sclerosis (ALS)

**Figure 1:**
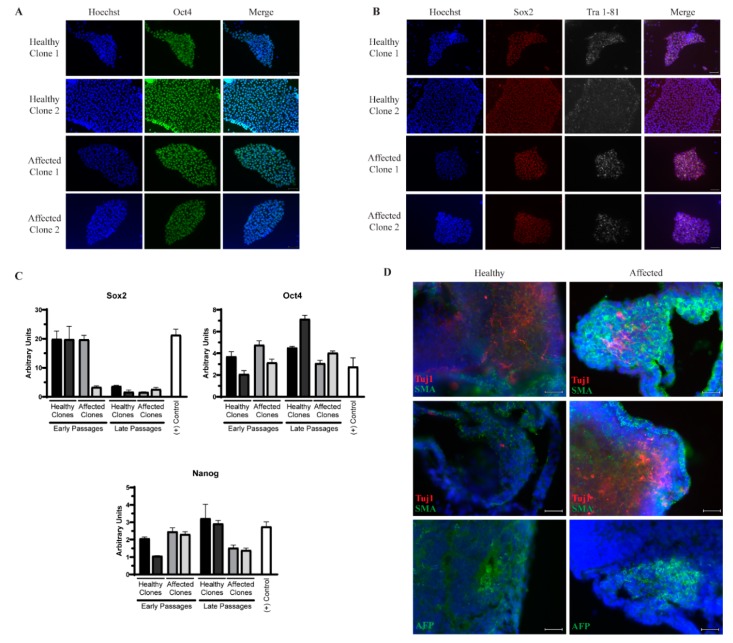
Successful reprogramming of induced pluripotent stem cells (iPSCs) from discordant identical twins. Two independent clones from each patient express the pluripotency markers Oct4 (**A**) and Sox2 and Tra 1-81 (**B**), detected by immunocytochemistry. Scale bar = 50 µm. (**C**) The transcript levels of Sox2, Oct4, and Nanog are similar to an established iPSC line by qPCR. (**D**) Embryoid body sections from each patient express markers of the three germ layers, including β-III tubulin (Tuj1, ectoderm), smooth muscle actin (SMA, mesoderm), and α-fetoprotein (AFP, endoderm) detected by immunocytochemistry. Scale bar = 25 µm.

We identified identical twin males who are discordant for ALS. The affected twin was diagnosed at age 59 with no family history of ALS and died at age 63. The unaffected individual is still ALS-free at the time of writing. Whole blood samples from both individuals were collected when the twins were 61 years old. Peripheral blood mononuclear cells (PBMCs) were then isolated and reprogrammed using Sendai virus expressing the Yamanaka factors [24]. Individual clones from each patient were expanded separately, and two clones from each were used for further experiments. The resulting iPSCs express the pluripotency markers Oct4, Sox2, and Nanog measured by qPCR (Figure 1C) and Oct4, Sox2, and Tra1-81 measured by immunocytochemistry (Figure 1A,B). Although there is a reduction in Sox2 transcript levels in later passages, this has not impacted the ability of the cells to expand as undifferentiated colonies or proceed through differentiation into MNs. Additionally, all three germ layers were generated by embryoid body formation as evidenced by positive staining for β-III tubulin (Tuj1) to mark ectoderm, α-fetoprotein (AFP) to mark endoderm, and smooth muscle actin (SMA) to mark mesoderm (Figure 1D), demonstrating sufficient reprogramming. 

### 3.2. Whole Genome Sequencing (WGS) Reveals no Meaningful Genetic Differences

Sporadic ALS (sALS) generally lacks known genetic mutations; however, de novo mutations in known ALS-causing genes have occurred in sporadic patients [1]. We therefore performed WGS on DNA isolated from whole blood. We compared the sequencing results to genes listed in ALS Online Database (ALSoD) (Table 2) and found a missense mutation in optineurin (*OPTN*). However, this particular variant has been linked to open-angle glaucoma [25] and is found in over 99% of samples deposited into gnomAD, a database of over 100,000 healthy whole genome and whole exome samples. We also found a 3′ UTR variant in *SIGMAR1*. However, mutations in *SIGMAR1* are linked to juvenile-onset ALS, so we speculate that this mutation is likely not disease-causing in these patients. Additionally, we found two missense mutations in chromogranin B (*CHGB*) that have been linked to Schizophrenia [26,27]. Finally, we found a small alanine duplication in *NIPA1*, a known risk allele for ALS [28]. However, as this mutation is shared between the twins, we do not believe that it is the primary driver of their discordance. We found no other known pathogenic or likely pathogenic mutations in any other ALS-causative gene, suggesting that these samples most likely represent a sporadic condition. 

### 3.3. Induced Pluripotent Stem Cells (iPSC)-Derived Motor Neurons (MNs) Have Similar Levels of Insoluble Proteins while Maintaining Viability

**Figure 2:**
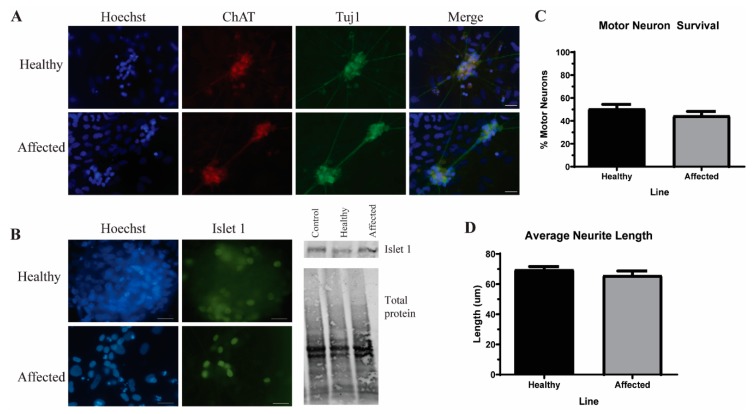
iPSC-derived motor neurons (MNs) from discordant identical twins show no viability or morphological differences. (**A**) Representative images of MNs from healthy and affected lines stained for ChAT (red) and Tuj1 (green). Scale bar = 25 µm. (**B**) Representative images show nuclear expression of the MN marker islet 1 (green) in both healthy and affected MN cultures. Western Blot for islet 1 also shows similar protein expression across the healthy and affected MN cultures. An unrelated control line was used as an additional control. Protein was normalized to total protein. (**C**) The quantification of A shows no difference in the number of ChAT+ MNs at four weeks of total differentiation. n = 3, not significant by Student’s *t*-test. (**D**) There is no significant alteration in the length of the neurites at 4 weeks of total differentiation. n = 3, not significant by Student’s *t*-test.

**Figure 3:**
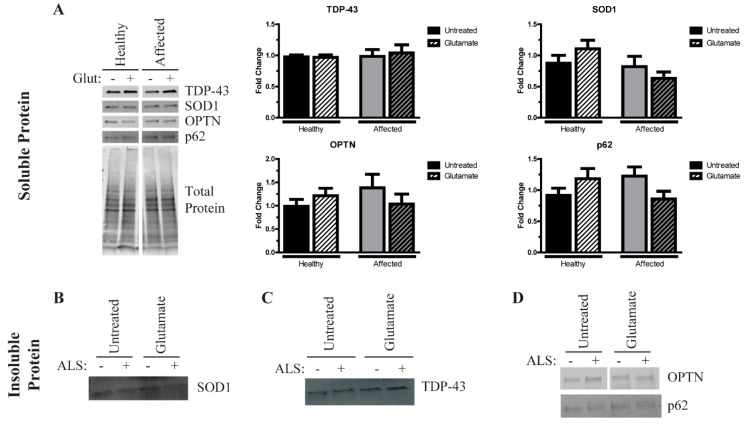
MNs from the discordant identical twins have similar levels of insoluble protein. (**A**) Western Blots of soluble protein samples show similar levels of SOD1, optineurin (OPTN), p62, or TDP-43 in the affected and unaffected twin MNs at baseline or after treatment with 100 µM glutamate for 96 h. n = 4–7. (**B**) There is no appreciable difference in the levels of insoluble SOD1 in the affected MNs compared to unaffected MNs at baseline. Treatment with 100 µM glutamate has a variable effect on aggregation in both cultures. n = 4–7. (**C**) Insoluble TDP-43 is similar in the affected and unaffected MNs that does not consistently increase or decrease after treatment with 100 µM glutamate. n = 4–7. (**D**) The levels of insoluble OPTN and p62 in the affected MNs are not significantly different compared to the unaffected MNs. Glutamate treatment has no consistent effect on insolubility. n = 4–7.

We next differentiated these iPSCs into MNs to determine whether any phenotypic alterations existed between the affected and unaffected samples. Using an established MN protocol [23], we found no significant difference in the number of ChAT+ MNs (Figure 2B) or in neurite length (Figure 2C), indicating no gross morphological or viability differences between the affected and unaffected MNs. As protein aggregation is a common feature of ALS, we next measured the level of insoluble proteins in these MNs. While small insoluble protein species of SOD1 (Figure 3B), TDP-43 (Figure 3C), OPTN, and p62 (Figure 3D) are detectable in the affected MN cultures, the levels are not significantly elevated compared to the unaffected twin. These data indicate that iPSC-derived MNs from the affected twin are not significantly different compared with the cultures from the discordant identical twin at baseline.

### 3.4. Excess Glutamate Increases the Insolubility of Aggregation-Prone Proteins

Since there were no overt phenotypes in the affected MN cultures compared to the unaffected control and baseline, we tested how the MNs reacted to exogenous stress. There are several non-cell autonomous contributors to ALS pathogenesis, and glutamate excitotoxicity is considered one of the prominent features [29]. We therefore treated healthy and affected MNs with 100 µM glutamate for a total of 96 h and determined their aggregation phenotype. Although others have observed fewer GFP-expressing iPSC-derived MNs following glutamate treatment [30], we did not observe a negative impact on cell viability or morphology for either the affected or unaffected twin (data not shown). Similarly, excess glutamate did not yield a consistent effect on the levels of insoluble protein (Figure 3). We found evidence of both increasing, unchanged, and decreased levels of insoluble proteins across the performed replicates, indicating that the stress paradigm tested here is not sufficient to yield a robust, consistent phenotype in MN cultures from discordant identical twins.

**Figure 4:**
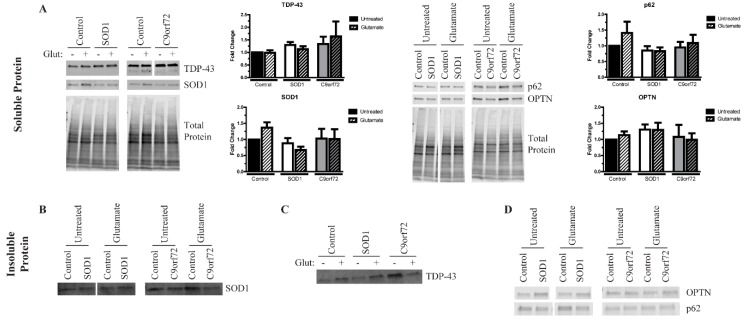
SOD1 and C9orf72 motor neurons have variable levels of insoluble protein. (**A**) SOD1 and C9orf72 MNs have similar levels of soluble SOD1, TDP-43, OPTN, and p62 compared to controls. n = 5–7. (**B**) Insoluble SOD1 is elevated primarily in SOD1 MNs, but not in C9orf72 MNs. These levels do not change after treatment with 100 µM glutamate. n = 5–7. (**C**) Western Blot of insoluble TDP-43 shows increased aggregation in SOD1 and C9orf72 MNs. SOD1 and control MNs tend to increase insoluble levels after glutamate stress, while the levels in C9orf72 MNs tend to decrease. n = 5–7. (**D**) SOD1 MNs tend to show higher levels of insoluble OPTN, whereas C9orf72 MNs show no apparent difference from controls. Insoluble p62 levels are unchanged in SOD1 and C9orf72 MNs compared to controls. n = 5–7.

Consistent with our previous results [15], SOD1 and C9orf72 iPSC-derived MNs tended to exhibit increased levels of insoluble proteins at baseline compared to control MNs (Figure 4), and we found higher levels of insoluble TDP-43 in SOD1 and C9orf72 MNs compared to controls at baseline (Figure 4C). Similar to the twin samples, neither soluble OPTN nor soluble p62 were different across the fALS MNs compared to control MNs (Figure 4A). Additionally, while the soluble levels of TDP-43 are unchanged in fALS cultures, an approximately 35 kDa soluble fragment is observed at higher exposures, which has been shown to be a more toxic form of TDP-43 [31,32,33,34] (Figure 4A). This soluble fragment can be detected in the C9orf72 MNs and occasionally observed in SOD1 MNs, which suggests that iPSCs recapitulate some important disease-relevant features. Of note, the 35 kDa TDP-43 fragment was also observed in the insoluble fraction in C9orf72 MNs, although it was less consistent than in the soluble fraction (data not shown). Insoluble SOD1 is slightly elevated primarily in SOD1 MNs, but this same trend was not observed in C9orf72 MNs (Figure 4B). Insoluble OPTN levels tended to increase in SOD1 MNs but not in C9orf72 MNs (Figure 4D). Levels of insoluble p62 are not appreciably different in fALS MNs compared to control MNs (Figure 4D). Together, these data indicate that fALS MNs exhibit some protein aggregation phenotypes similar to what is seen in post-mortem samples. 

We next tested the impact of excess glutamate on fALS iPSC-derived motor neurons. Like the MNs derived from the identical twins, a 96-h treatment with 100 µM glutamate did not alter overall morphology or viability in mutant SOD1, C9orf72, and control iPSC-derived MNs (data not shown). Yet, this treatment was sufficient to induce a consistent change in insoluble TDP-43 protein levels (Figure 4C). Both the control and SOD1 MNs had higher levels of insoluble TDP-43 after glutamate treatment, although glutamate treatment had little effect on the expression of the TDP-43 fragment (data not shown). Remarkably, the C9orf72 MNs, in contrast, had reduced levels of full-length insoluble TDP-43 after the same stress, which could account for the modest increase in soluble TDP-43. Regarding SOD1 aggregation, the SOD1 MNs had modestly reduced levels of soluble SOD1 after glutamate treatment (Figure 4A), but this did not translate to higher levels of insoluble SOD1 (Figure 4B), suggesting that SOD1 may instead be degraded under this stress. In contrast, glutamate treatment did not alter soluble or insoluble SOD1 levels in the C9orf72 MNs. Finally, insoluble levels of neither OPTN nor p62 were affected by glutamate treatment. Together, these data indicate that excess glutamate does not cause a consistent effect on protein insolubility, suggesting that the mechanisms leading to protein aggregation may be distinct for each protein and genetic background. 

### 3.5. Alterations in Autophagic Flux in Amyotrophic Lateral Sclerosis (ALS) Motor Neurons (MNs)

**Figure 5:**
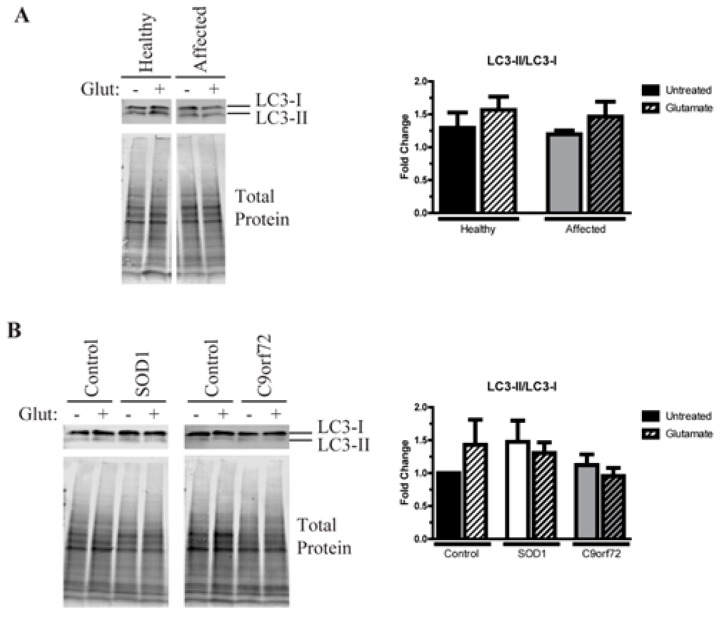
Alterations in autophagy in familial ALS (fALS) motor neurons. (**A**) The Western blot for LC3 shows similar levels of autophagy in affected and unaffected MNs at baseline, and MNs from both twins increase autophagic flux after treatment with 100 µM glutamate for 96 h. n = 4–7. (**B**) SOD1 MNs have an increase in the LC3-II/LC3-I ratio at baseline compared to control MNs, indicating a higher autophagic flux in these cells. Control MNs increase autophagy after a 96-h treatment with 100 µM glutamate, while the SOD1 and C9orf72 MNs do not. n = 5–7.

Since autophagy is a common mechanism by which a cell degrades misfolded and aggregated proteins, we next tested whether autophagic flux was disrupted in ALS iPSC-derived MNs. We found that the ratio of LC3-II/LC3-I is not changed in the affected MNs compared to the unaffected twin at baseline but does increase after glutamate treatment (Figure 5A). This suggests that MN cultures from both twins increase autophagic flux in response to exogenous stress as a potential compensatory mechanism to guard against protein aggregation. Similarly, control MNs from other unrelated individuals also activate autophagy after glutamate stress (Figure 5B). However, while SOD1 MNs have a higher LC3-II/LC3-1 ratio at baseline compared to control MNs (Figure 5B), neither these MNs nor the C9orf72 MNs increase autophagic flux in response to excess glutamate. Together, these data suggest that fALS MNs do not efficiently increase autophagy in response to insoluble protein burden or glutamate toxicity, suggesting poor regulation of protective mechanisms designed to counteract insoluble protein aggregation.

## 4. Discussion

Induced pluripotent stem cells (iPSCs) are a powerful modeling tool to study both sporadic and genetically linked disease. Still, few studies have generated iPSC lines from sALS patients [35,36,37,38] despite the abundance of sALS compared to fALS. Given the heterogeneity in the causes of fALS, it is unclear how accurately these models mimic the sporadic condition. Additionally, the inability to generate isogenic control iPSC lines for sALS patients is an experimental limitation, which makes generating iPSC lines from discordant identical twins a truly valuable resource.

While sALS is generally believed to occur in the absence of genetic mutations, previous data have found de novo mutations in sporadic patients [1]. Still, given the lack of family history of the disease, as well as the continued discordance between the identical twins, we did not expect to find mutations in known ALS genes. Indeed, WGS revealed no meaningful variants in any gene found in ALSoD (Table 2). However, given the observed discordance between the brothers, the affected twin likely has either a novel de novo mutation(s) that contributed to his disease, or experienced an environmental stressor that his brother did not. We unfortunately have no further clinical data on the brothers’ lives, leaving the environmental question unanswered. However, further analysis on the WGS data could be done to identify any potential novel variants. 

Like most MNs derived from ALS iPSC lines [39], the affected MNs presented here do not exhibit an overall reduction of viability or gross morphological alterations (Figure 2B,C). While divergent from the disease symptomology, iPSC-derived neurons are most like fetal neurons due to the epigenetic reversal of aging marks during the reprogramming process [40,41] and therefore tend to not have viability problems [39]. However, as protein aggregation is the pathological hallmark of ALS that has been observed in iPSC models [39], we measured the levels of insoluble protein to determine whether these iPSC lines were showing disease-relevant phenotypes while maintaining viability. Typically, TDP-43 is aggregated, although OPTN, p62, ubiquitin, and SOD1 can also be found in inclusions in post-mortem tissues [3,4,5]. We detected insoluble protein consistent with an aggregation phenotype in the affected twin MNs. However, these levels were not enhanced compared to the unaffected twin cultures (Figure 3B–D). Similarly, although excess glutamate had a consistent effect on TDP-43 aggregation in the independent control, SOD1, and C9orf72 iPSC-derived MNs, the same stressor did not yield consistent alterations on protein solubility in either the affected or unaffected twin MN cultures. Therefore, the iPSC-derived MNs from the discordant identical twins are largely phenotypically identical, suggesting that their discordance may have been due to an epigenetic variation that was erased during the reprogramming process. 

Consistent with our previous work [15], we found an increased presence of insoluble proteins in MN cultures derived from SOD1 and C9orf72 iPSC lines compared to independent control lines (Figure 4B–D). Previous data have suggested that SOD1 and TDP-43 aggregation are distinct events [3]. However, the data presented here and previously suggest that this distinction may not be as simple [15]. We observed SOD1 aggregates in the SOD1 MNs (Figure 4B), but we found that both SOD1 and C9orf72 MNs exhibit higher levels of TDP-43 aggregation compared to controls (Figure 4C). Together with higher levels of insoluble OPTN (Figure 4D), fALS MNs may follow a similar, yet complex aggregation mechanism involving many different proteins. 

Interestingly, we also observed a 35 kDa fragment of TDP-43 within the soluble fraction in the C9orf72 MNs (Figure 4A). Evidence suggests that the N-terminal truncation of TDP-43 via alternative splicing generates the 35 kDa fragment [33,42]. This cleavage product has been associated with a more toxic aggregation phenotype as it primarily localizes in the cytoplasm and acts as a seed to promote further accumulation of both full-length and fragmented TDP-43 [31,32]. While this fragment has been reported in patient post-mortem samples [32,33], this is the first report of the fragmented species occurring spontaneously within iPSC-derived MNs. Although additional research and validation is needed, these data suggest that C9orf72 iPSC MNs may recapitulate some of the key TDP-43 alternative splicing events. Together with the aggregation phenotype, these data further indicate that iPSC-derived MNs from fALS patients exhibit some of the intricate disease phenotypes despite maintaining their viability. It remains to be determined how this fragment could contribute to downstream disease pathology, but the ALS iPSC model system may be a valuable resource to investigate this question. 

While the field has largely taken a MN-centric view of ALS, there are undoubtably multifaceted interactions between additional cell types within the brain and spinal cord of ALS patients. Indeed, it is well established that astrocytes can drive disease pathogenesis [43]. Although some studies have aimed to identify secreted factors released by the astrocytes that contribute to MN death [44,45,46], few have assessed the sublethal changes in MNs that may promote downstream malfunction and loss. Excess glutamate in the synapses that persists due to a reduced uptake by the astrocytes [47,48] is a proposed mechanism leading to MN loss in ALS. However, as clinical symptoms of ALS do not typically manifest until middle age, it is likely that sublethal stressors are impacting the nervous system for many years prior to clinical onset. However, very little is known about what cellular and molecular effects sublethal glutamate exposure has on MNs. A 96-h treatment with 100 µM glutamate did not induce morphological or viability deficits in any of the iPSC-derived MNs studied here (data not shown) but had a variable effect on protein aggregation. Glutamate seemed to have the most robust effect on TDP-43 aggregation in the fALS MNs, leading to increased levels in the independent controls and SOD1 MNs but decreased levels in the C9orf72 MNs (Figure 4C). The reason for this discordance is not immediately clear, as neither the SOD1 nor C9orf72 MNs increase autophagy after this treatment, suggesting that the degradation of aggregates is not increasing in the C9orf72 MNs. However, it is possible that this decrease is indicative of TDP-43 transitioning to a more toxic, phase-separated state in these MNs, as has been suggested by a recent publication [12]. Protein aggregation is just one of many potential disease properties, so additional work is needed to determine how sublethal stress affects such properties as mitochondrial function, axonal trafficking, and synaptic function. Moreover, because the iPSCs can be coaxed into the various disease-relevant tissues, including MNs, astrocytes, and muscle cells, it will be important to utilize these newly generated iPSCs to further investigate the molecular and cellular functions and interactions to get a more comprehensive view of disease processes. 

The iPSC-derived MNs from discordant identical twins seem to exist in a phenotypic space between “ALS-like” and “control-like”. MNs from both twins exhibit evidence of protein aggregation that are not significantly different from each other but do appear higher than independent controls (Figure 3 and Figure 4). Additionally, affected and unaffected MNs increase autophagy after glutamate stress at a similar magnitude to independent controls, while SOD1 and C9orf72 MNs do not (Figure 5). This suggests that the identical twins may share a previously unidentified risk allele(s) that made them equally susceptible to the disease, but that the affected twin developed ALS due to other unknown risks or stressors. Additionally, the difference in phenotypes between the affected twin and fALS MNs indicate that sporadic and familial ALS may have distinct pathologies, and therefore may not respond to the same therapy. Further studies are needed to determine whether this difference is unique to the sporadic lines described here or common to multiple sporadic forms of the disease. 

Together, these data suggest that glutamate may contribute to protein aggregation in a protein- and genotype-dependent manner. Moreover, sALS and fALS may follow different pathological mechanisms and should be separated into distinct subgroups when developing and validating new therapies. As such, the iPSC lines from discordant identical twins generated in this study will be a valuable tool for studying sALS in an isogenic manner in order to better understand the phenotypes and pathological mechanisms in ALS. 

## Figures and Tables

**Table 1:** Primary antibodies.

Antibody	Company	Catalog Number
Goat α-ChAT	Millipore	AB144P
Rabbit α-LC3	Cell Signaling	3868
Rabbit α-Optineurin	abcam	ab151240
Mouse α-p62	BD Biosciences	610832
Rabbit α-SOD1	abcam	ab13498
Rabbit α-TDP-43	abcam	ab109535
Rabbit α-Tuj1	Covance	MRB-435P
Mouse Islet1	DSHB	40.2D6

**Table 2:** Abridged list of identified variants in genes listed in Amyotrophic Lateral Sclerosis Online Database (ALSoD).

Gene	Mutation	Genotype (Affected/ Unaffected)	Variant Type	Clinical Significance	gnomAD Frequency	HGMD Phenotype
CHGB	c.1058C>G p.Ala353Gly	Heterozygous/ Heterozygous	Missense	Variant of Unknown Significance	0.451	Schizophrenia
CHGB	c.1250G>A p.Arg417His	Heterozygous/ Heterozygous	Missense	Variant of Unknown Significance	0.277	Schizophrenia
OPTN	c.964A>G p.Lys322Glut	Homozygous/ Homozygous	Missense	Pathogenic	0.997	Open Angle Glaucoma
SIGMAR1	c.*31A>G	Homozygous/ Homozygous	3′ UTR variant	Variant of Unknown Significance	0.995	Amyotrophic lateral sclerosis
NIPA1	c.42_47dupGGCGGC p.Ala15_Ala16dup	Heterozygous/ Heterozygous	Disruptive inframe insertion	Variant of Unknown Significance	-	-
